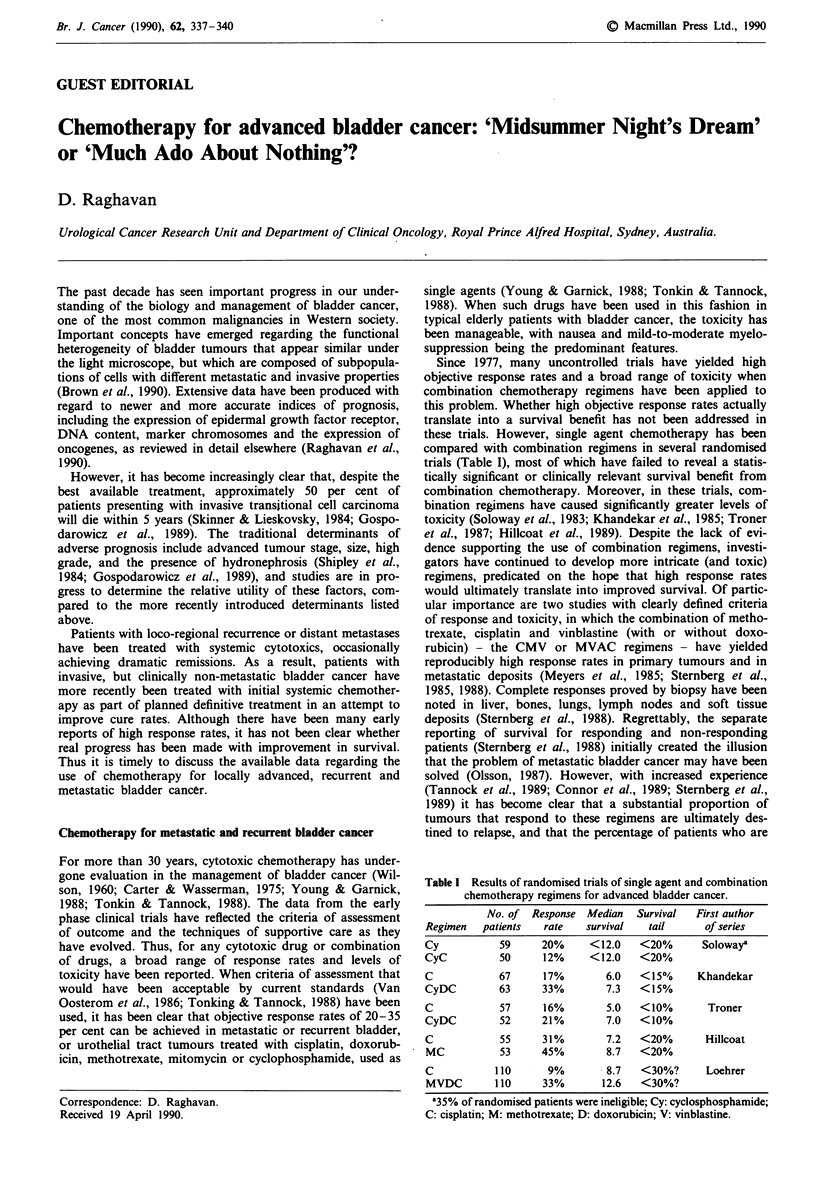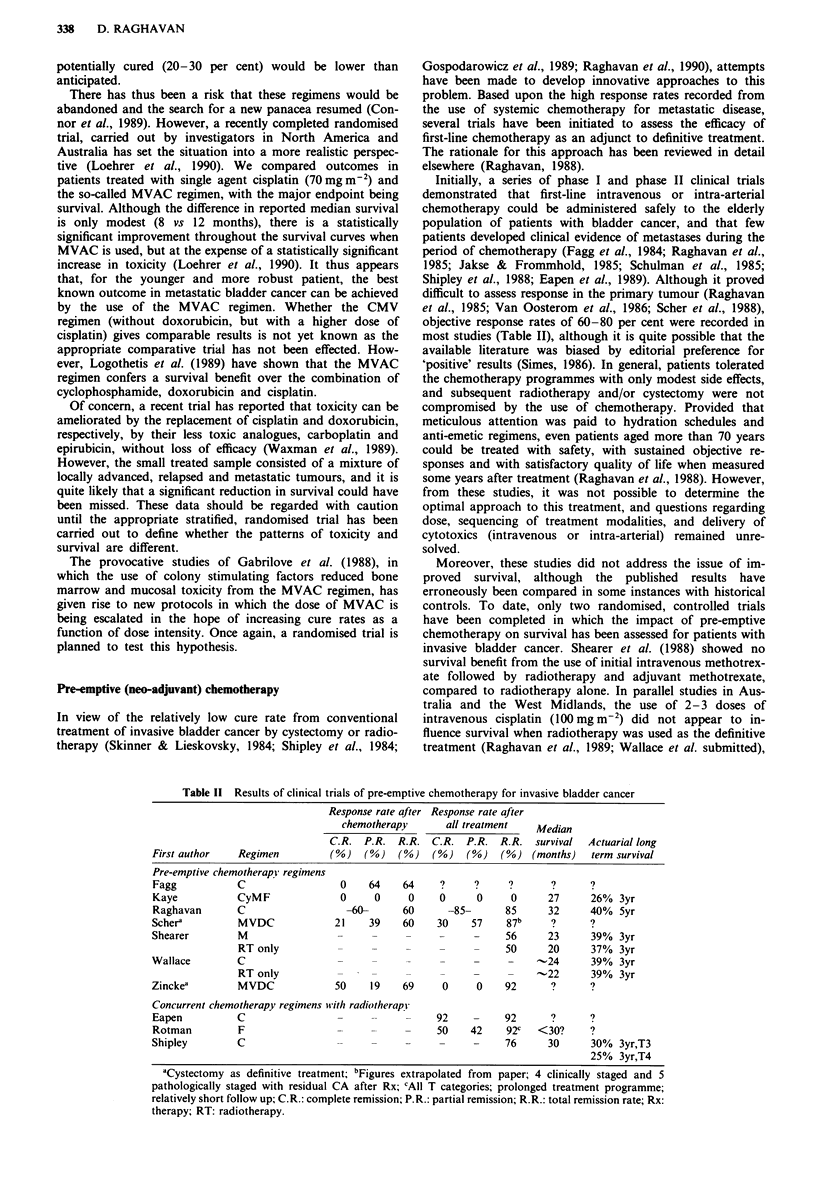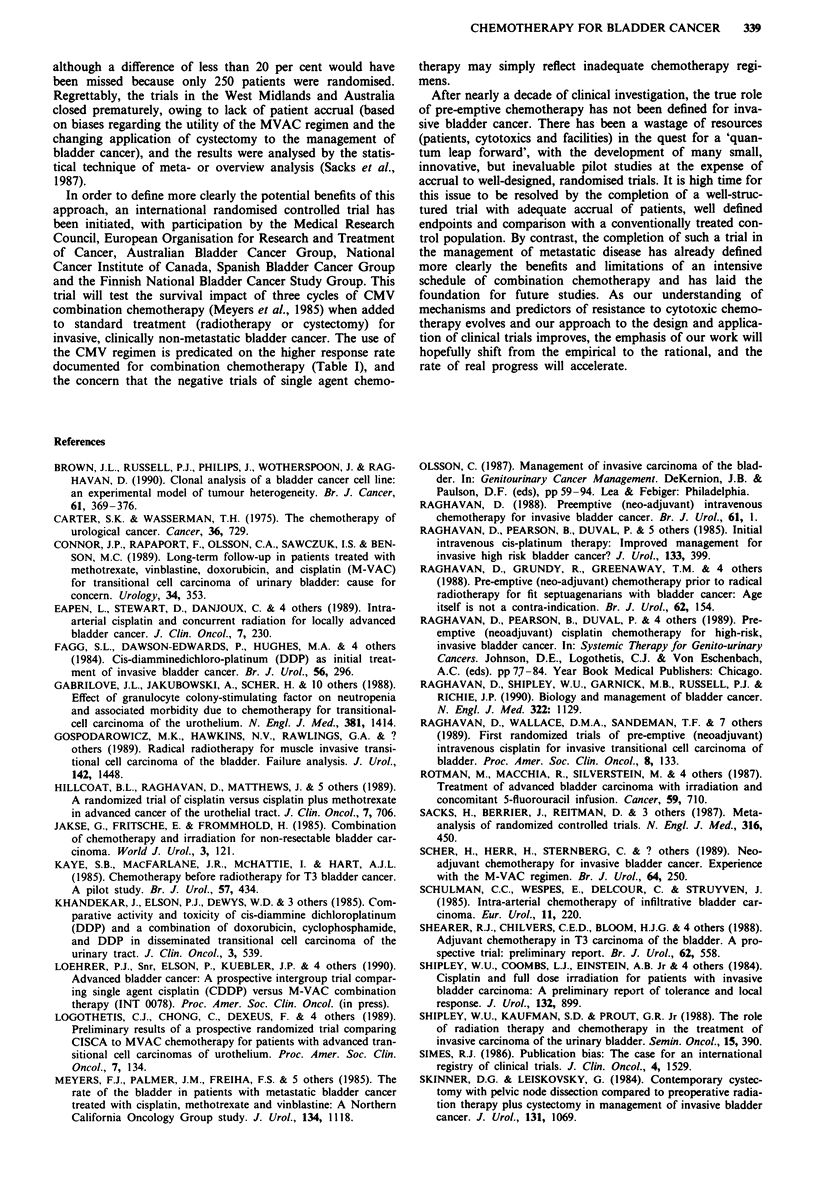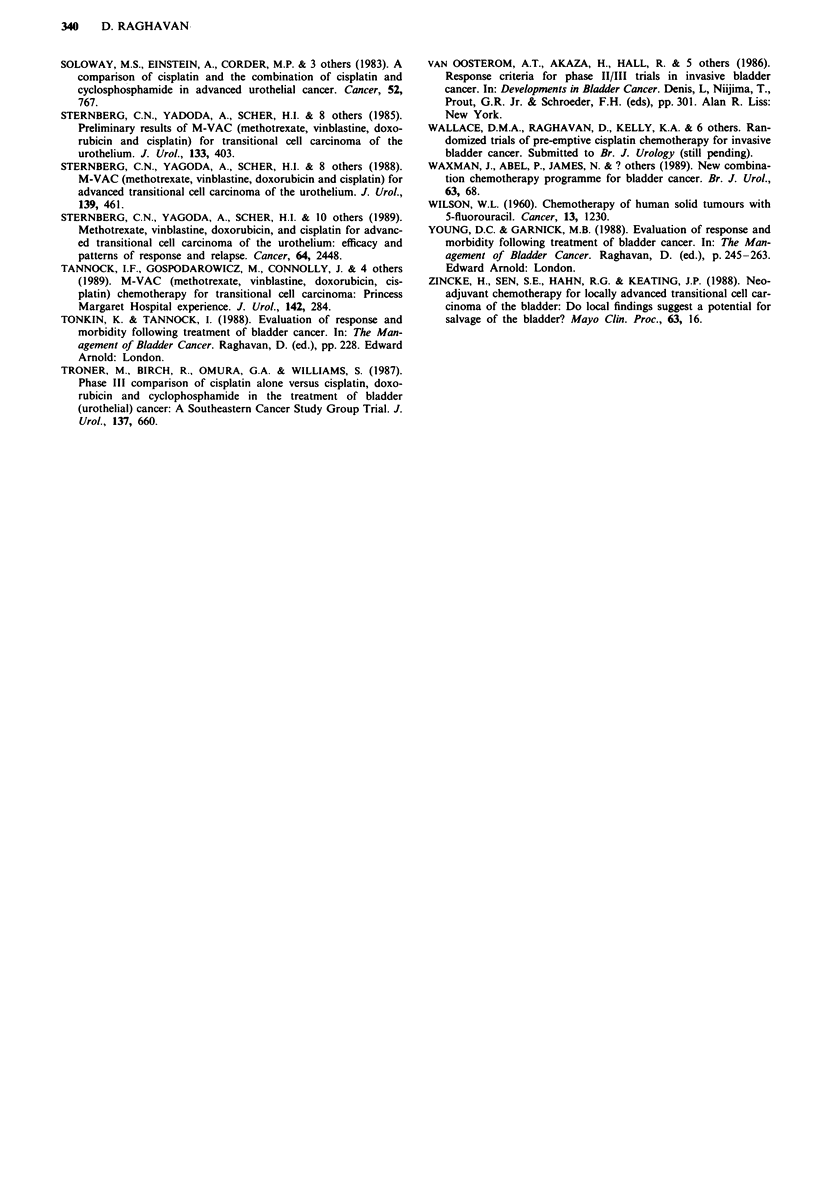# Chemotherapy for advanced bladder cancer: 'Midsummer Night's Dream' or 'Much Ado About Nothing'?

**DOI:** 10.1038/bjc.1990.293

**Published:** 1990-09

**Authors:** D. Raghavan

**Affiliations:** Urological Cancer Research Unit, Royal Prince Alfred Hospital, Sydney, Australia.


Br. J. Cancer (1990), 62, 337-340 © Macmillan Press Ltd., 1990

GUEST EDITORIAL

### Chemotherapy for advanced bladder cancer: 'Midsummer Night's Dream' or 'Much Ado About Nothing'?

D. Raghavan

*Urological Cancer Research Unit and Department of Clinical Oncology, Royal Prince Alfred Hospital, Sydney, Australia.*

The past decade has seen important progress in our understanding of the biology and management of bladder cancer, one of the most common malignancies in Western society. Important concepts have emerged regarding the functional heterogeneity of bladder tumours that appear similar under the light microscope, but which are composed of subpopulations of cells with different metastatic and invasive properties (Brown *et al.*, 1990). Extensive data have been produced with regard to newer and more accurate indices of prognosis, including the expression of epidermal growth factor receptor, DNA content, marker chromosomes and the expression of oncogenes, as reviewed in detail elsewhere (Raghavan *et al.*, 1990).

However, it has become increasingly clear that, despite the best available treatment, approximately 50 per cent of patients presenting with invasive transitional cell carcinoma will die within 5 years (Skinner & Lieskovsky, 1984; Gospodarowicz *et al.*, 1989). The traditional determinants of adverse prognosis include advanced tumour stage, size, high grade, and the presence of hydronephrosis (Shipley *et al.*, 1984; Gospodarowicz *et al.*, 1989), and studies are in progress to determine the relative utility of these factors, compared to the more recently introduced determinants listed above.

Patients with loco-regional recurrence or distant metastases have been treated with systemic cytotoxics, occasionally achieving dramatic remissions. As a result, patients with invasive, but clinically non-metastatic bladder cancer have more recently been treated with initial systemic chemotherapy as part of planned definitive treatment in an attempt to improve cure rates. Although there have been many early reports of high response rates, it has not been clear whether real progress has been made with improvement in survival. Thus it is timely to discuss the available data regarding the use of chemotherapy for locally advanced, recurrent and metastatic bladder cancer.

### Chemotherapy for metastatic and recurrent bladder cancer

For more than 30 years, cytotoxic chemotherapy has undergone evaluation in the management of bladder cancer (Wilson, 1960; Carter & Wasserman, 1975; Young & Garnick, 1988; Tonkin & Tannock, 1988). The data from the early phase clinical trials have reflected the criteria of assessment of outcome and the techniques of supportive care as they have evolved. Thus, for any cytotoxic drug or combination of drugs, a broad range of response rates and levels of toxicity have been reported. When criteria of assessment that would have been acceptable by current standards (Van Oosterom *et al.*, 1986; Tonking & Tannock, 1988) have been used, it has been clear that objective response rates of 20-35 per cent can be achieved in metastatic or recurrent bladder, or urothelial tract tumours treated with cisplatin, doxorubicin, methotrexate, mitomycin or cyclophosphamide, used as single agents (Young & Garnick, 1988; Tonkin & Tannock, 1988). When such drugs have been used in this fashion in typical elderly patients with bladder cancer, the toxicity has been manageable, with nausea and mild-to-moderate myelosuppression being the predominant features.

Since 1977, many uncontrolled trials have yielded high objective response rates and a broad range of toxicity when combination chemotherapy regimens have been applied to this problem. Whether high objective response rates actually translate into a survival benefit has not been addressed in these trials. However, single agent chemotherapy has been compared with combination regimens in several randomised trials (Table I), most of which have failed to reveal a statistically significant or clinically relevant survival benefit from combination chemotherapy. Moreover, in these trials, combination regimens have caused significantly greater levels of toxicity (Soloway *et al.*, 1983; Khandekar *et al.*, 1985; Troner *et al.*, 1987; Hillcoat *et al.*, 1989). Despite the lack of evidence supporting the use of combination regimens, investigators have continued to develop more intricate (and toxic) regimens, predicated on the hope that high response rates would ultimately translate into improved survival. Of particular importance are two studies with clearly defined criteria of response and toxicity, in which the combination of methotrexate, cisplatin and vinblastine (with or without doxorubicin) – the CMV or MVAC regimens – have yielded reproducibly high response rates in primary tumours and in metastatic deposits (Meyers *et al.*, 1985; Sternberg *et al.*, 1985, 1988). Complete responses proved by biopsy have been noted in liver, bones, lungs, lymph nodes and soft tissue deposits (Sternberg *et al.*, 1988). Regrettably, the separate reporting of survival for responding and non-responding patients (Sternberg *et al.*, 1988) initially created the illusion that the problem of metastatic bladder cancer may have been solved (Olsson, 1987). However, with increased experience (Tannock *et al.*, 1989; Connor *et al.*, 1989; Sternberg *et al.*, 1989) it has become clear that a substantial proportion of tumours that respond to these regimens are ultimately destined to relapse, and that the percentage of patients who are

**Table I** Results of randomised trials of single agent and combination chemotherapy regimens for advanced bladder cancer.

| *Regimen* | *No. of patients* | *Response rate* | *Median survival* | *Survival tail* | *First author of series* |
|---|---|---|---|---|---|
| Cy | 59 | 20% | <12.0 | <20% | Soloway[a] |
| CyC | 50 | 12% | <12.0 | <20% | |
| C | 67 | 17% | 6.0 | <15% | Khandekar |
| CyDC | 63 | 33% | 7.3 | <15% | |
| C | 57 | 16% | 5.0 | <10% | Troner |
| CyDC | 52 | 21% | 7.0 | <10% | |
| C | 55 | 31% | 7.2 | <20% | Hillcoat |
| MC | 53 | 45% | 8.7 | <20% | |
| C | 110 | 9% | 8.7 | <30%? | Loehrer |
| MVDC | 110 | 33% | 12.6 | <30%? | |

[a]35% of randomised patients were ineligible; Cy: cyclosphosphamide; C: cisplatin; M: methotrexate; D: doxorubicin; V: vinblastine.

Correspondence: D. Raghavan.
Received 19 April 1990.

potentially cured (20–30 per cent) would be lower than anticipated.

There has thus been a risk that these regimens would be abandoned and the search for a new panacea resumed (Connor *et al.*, 1989). However, a recently completed randomised trial, carried out by investigators in North America and Australia has set the situation into a more realistic perspective (Loehrer *et al.*, 1990). We compared outcomes in patients treated with single agent cisplatin ($70\,mg\,m^{-2}$) and the so-called MVAC regimen, with the major endpoint being survival. Although the difference in reported median survival is only modest (8 *vs* 12 months), there is a statistically significant improvement throughout the survival curves when MVAC is used, but at the expense of a statistically significant increase in toxicity (Loehrer *et al.*, 1990). It thus appears that, for the younger and more robust patient, the best known outcome in metastatic bladder cancer can be achieved by the use of the MVAC regimen. Whether the CMV regimen (without doxorubicin, but with a higher dose of cisplatin) gives comparable results is not yet known as the appropriate comparative trial has not been effected. However, Logothetis *et al.* (1989) have shown that the MVAC regimen confers a survival benefit over the combination of cyclophosphamide, doxorubicin and cisplatin.

Of concern, a recent trial has reported that toxicity can be ameliorated by the replacement of cisplatin and doxorubicin, respectively, by their less toxic analogues, carboplatin and epirubicin, without loss of efficacy (Waxman *et al.*, 1989). However, the small treated sample consisted of a mixture of locally advanced, relapsed and metastatic tumours, and it is quite likely that a significant reduction in survival could have been missed. These data should be regarded with caution until the appropriate stratified, randomised trial has been carried out to define whether the patterns of toxicity and survival are different.

The provocative studies of Gabrilove *et al.* (1988), in which the use of colony stimulating factors reduced bone marrow and mucosal toxicity from the MVAC regimen, has given rise to new protocols in which the dose of MVAC is being escalated in the hope of increasing cure rates as a function of dose intensity. Once again, a randomised trial is planned to test this hypothesis.

### Pre-emptive (neo-adjuvant) chemotherapy

In view of the relatively low cure rate from conventional treatment of invasive bladder cancer by cystectomy or radiotherapy (Skinner & Lieskovsky, 1984; Shipley *et al.*, 1984; Gospodarowicz *et al.*, 1989; Raghavan *et al.*, 1990), attempts have been made to develop innovative approaches to this problem. Based upon the high response rates recorded from the use of systemic chemotherapy for metastatic disease, several trials have been initiated to assess the efficacy of first-line chemotherapy as an adjunct to definitive treatment. The rationale for this approach has been reviewed in detail elsewhere (Raghavan, 1988).

Initially, a series of phase I and phase II clinical trials demonstrated that first-line intravenous or intra-arterial chemotherapy could be administered safely to the elderly population of patients with bladder cancer, and that few patients developed clinical evidence of metastases during the period of chemotherapy (Fagg *et al.*, 1984; Raghavan *et al.*, 1985; Jakse & Frommhold, 1985; Schulman *et al.*, 1985; Shipley *et al.*, 1988; Eapen *et al.*, 1989). Although it proved difficult to assess response in the primary tumour (Raghavan *et al.*, 1985; Van Oosterom *et al.*, 1986; Scher *et al.*, 1988), objective response rates of 60–80 per cent were recorded in most studies (Table II), although it is quite possible that the available literature was biased by editorial preference for 'positive' results (Simes, 1986). In general, patients tolerated the chemotherapy programmes with only modest side effects, and subsequent radiotherapy and/or cystectomy were not compromised by the use of chemotherapy. Provided that meticulous attention was paid to hydration schedules and anti-emetic regimens, even patients aged more than 70 years could be treated with safety, with sustained objective responses and with satisfactory quality of life when measured some years after treatment (Raghavan *et al.*, 1988). However, from these studies, it was not possible to determine the optimal approach to this treatment, and questions regarding dose, sequencing of treatment modalities, and delivery of cytotoxics (intravenous or intra-arterial) remained unresolved.

Moreover, these studies did not address the issue of improved survival, although the published results have erroneously been compared in some instances with historical controls. To date, only two randomised, controlled trials have been completed in which the impact of pre-emptive chemotherapy on survival has been assessed for patients with invasive bladder cancer. Shearer *et al.* (1988) showed no survival benefit from the use of initial intravenous methotrexate followed by radiotherapy and adjuvant methotrexate, compared to radiotherapy alone. In parallel studies in Australia and the West Midlands, the use of 2–3 doses of intravenous cisplatin ($100\,mg\,m^{-2}$) did not appear to influence survival when radiotherapy was used as the definitive treatment (Raghavan *et al.*, 1989; Wallace *et al.* submitted),

**Table II** Results of clinical trials of pre-emptive chemotherapy for invasive bladder cancer

| | | *Response rate after chemotherapy* | | | *Response rate after all treatment* | | | | |
|---|---|---|---|---|---|---|---|---|---|
| *First author* | *Regimen* | *C.R. (%)* | *P.R. (%)* | *R.R. (%)* | *C.R. (%)* | *P.R. (%)* | *R.R. (%)* | *Median survival (months)* | *Actuarial long term survival* |
| *Pre-emptive chemotherapy regimens* | | | | | | | | | |
| Fagg | C | 0 | 64 | 64 | ? | ? | ? | ? | ? |
| Kaye | CyMF | 0 | 0 | 0 | 0 | 0 | 0 | 27 | 26% 3yr |
| Raghavan | C | –60– | | 60 | –85– | | 85 | 32 | 40% 5yr |
| Scher[a] | MVDC | 21 | 39 | 60 | 30 | 57 | 87[b] | ? | ? |
| Shearer | M | – | – | – | – | – | 56 | 23 | 39% 3yr |
| | RT only | – | – | – | – | – | 50 | 20 | 37% 3yr |
| Wallace | C | – | – | – | – | – | – | ~24 | 39% 3yr |
| | RT only | – | – | – | – | – | – | ~22 | 39% 3yr |
| Zincke[a] | MVDC | 50 | 19 | 69 | 0 | 0 | 92 | ? | ? |
| *Concurrent chemotherapy regimens with radiotherapy* | | | | | | | | | |
| Eapen | C | – | – | – | 92 | – | 92 | ? | ? |
| Rotman | F | – | – | – | 50 | 42 | 92[c] | <30? | ? |
| Shipley | C | – | – | – | – | – | 76 | 30 | 30% 3yr,T3<br>25% 3yr,T4 |

[a]Cystectomy as definitive treatment; [b]Figures extrapolated from paper; 4 clinically staged and 5 pathologically staged with residual CA after Rx; [c]All T categories; prolonged treatment programme; relatively short follow up; C.R.: complete remission; P.R.: partial remission; R.R.: total remission rate; Rx: therapy; RT: radiotherapy.

although a difference of less than 20 per cent would have been missed because only 250 patients were randomised. Regrettably, the trials in the West Midlands and Australia closed prematurely, owing to lack of patient accrual (based on biases regarding the utility of the MVAC regimen and the changing application of cystectomy to the management of bladder cancer), and the results were analysed by the statistical technique of meta- or overview analysis (Sacks *et al.*, 1987).

In order to define more clearly the potential benefits of this approach, an international randomised controlled trial has been initiated, with participation by the Medical Research Council, European Organisation for Research and Treatment of Cancer, Australian Bladder Cancer Group, National Cancer Institute of Canada, Spanish Bladder Cancer Group and the Finnish National Bladder Cancer Study Group. This trial will test the survival impact of three cycles of CMV combination chemotherapy (Meyers *et al.*, 1985) when added to standard treatment (radiotherapy or cystectomy) for invasive, clinically non-metastatic bladder cancer. The use of the CMV regimen is predicated on the higher response rate documented for combination chemotherapy (Table I), and the concern that the negative trials of single agent chemotherapy may simply reflect inadequate chemotherapy regimens.

After nearly a decade of clinical investigation, the true role of pre-emptive chemotherapy has not been defined for invasive bladder cancer. There has been a wastage of resources (patients, cytotoxics and facilities) in the quest for a 'quantum leap forward', with the development of many small, innovative, but inevaluable pilot studies at the expense of accrual to well-designed, randomised trials. It is high time for this issue to be resolved by the completion of a well-structured trial with adequate accrual of patients, well defined endpoints and comparison with a conventionally treated control population. By contrast, the completion of such a trial in the management of metastatic disease has already defined more clearly the benefits and limitations of an intensive schedule of combination chemotherapy and has laid the foundation for future studies. As our understanding of mechanisms and predictors of resistance to cytotoxic chemotherapy evolves and our approach to the design and application of clinical trials improves, the emphasis of our work will hopefully shift from the empirical to the rational, and the rate of real progress will accelerate.

### References

BROWN, J.L., RUSSELL, P.J., PHILIPS, J., WOTHERSPOON, J. & RAGHAVAN, D. (1990). Clonal analysis of a bladder cancer cell line: an experimental model of tumour heterogeneity. *Br. J. Cancer*, **61**, 369–376.

CARTER, S.K. & WASSERMAN, T.H. (1975). The chemotherapy of urological cancer. *Cancer*, **36**, 729.

CONNOR, J.P., RAPAPORT, F., OLSSON, C.A., SAWCZUK, I.S. & BENSON, M.C. (1989). Long-term follow-up in patients treated with methotrexate, vinblastine, doxorubicin, and cisplatin (M-VAC) for transitional cell carcinoma of urinary bladder: cause for concern. *Urology*, **34**, 353.

EAPEN, L., STEWART, D., DANJOUX, C. & 4 others (1989). Intra-arterial cisplatin and concurrent radiation for locally advanced bladder cancer. *J. Clin. Oncol.*, **7**, 230.

FAGG, S.L., DAWSON-EDWARDS, P., HUGHES, M.A. & 4 others (1984). Cis-diamminedichloro-platinum (DDP) as initial treatment of invasive bladder cancer. *Br. J. Urol.*, **56**, 296.

GABRILOVE, J.L., JAKUBOWSKI, A., SCHER, H. & 10 others (1988). Effect of granulocyte colony-stimulating factor on neutropenia and associated morbidity due to chemotherapy for transitional-cell carcinoma of the urothelium. *N. Engl. J. Med.*, **381**, 1414.

GOSPODAROWICZ, M.K., HAWKINS, N.V., RAWLINGS, G.A. & ? others (1989). Radical radiotherapy for muscle invasive transitional cell carcinoma of the bladder. Failure analysis. *J. Urol.*, **142**, 1448.

HILLCOAT, B.L., RAGHAVAN, D., MATTHEWS, J. & 5 others (1989). A randomized trial of cisplatin versus cisplatin plus methotrexate in advanced cancer of the urothelial tract. *J. Clin. Oncol.*, **7**, 706.

JAKSE, G., FRITSCHE, E. & FROMMHOLD, H. (1985). Combination of chemotherapy and irradiation for non-resectable bladder carcinoma. *World J. Urol.*, **3**, 121.

KAYE, S.B., MACFARLANE, J.R., MCHATTIE, I. & HART, A.J.L. (1985). Chemotherapy before radiotherapy for T3 bladder cancer. A pilot study. *Br. J. Urol.*, **57**, 434.

KHANDEKAR, J., ELSON, P.J., DEWYS, W.D. & 3 others (1985). Comparative activity and toxicity of cis-diammine dichloroplatinum (DDP) and a combination of doxorubicin, cyclophosphamide, and DDP in disseminated transitional cell carcinoma of the urinary tract. *J. Clin. Oncol.*, **3**, 539.

LOEHRER, P.J., Snr, ELSON, P., KUEBLER, J.P. & 4 others (1990). Advanced bladder cancer: A prospective intergroup trial comparing single agent cisplatin (CDDP) versus M-VAC combination therapy (INT 0078). *Proc. Amer. Soc. Clin. Oncol.* (in press).

LOGOTHETIS, C.J., CHONG, C., DEXEUS, F. & 4 others (1989). Preliminary results of a prospective randomized trial comparing CISCA to MVAC chemotherapy for patients with advanced transitional cell carcinomas of urothelium. *Proc. Amer. Soc. Clin. Oncol.*, **7**, 134.

MEYERS, F.J., PALMER, J.M., FREIHA, F.S. & 5 others (1985). The rate of the bladder in patients with metastatic bladder cancer treated with cisplatin, methotrexate and vinblastine: A Northern California Oncology Group study. *J. Urol.*, **134**, 1118.

OLSSON, C. (1987). Management of invasive carcinoma of the bladder. In: *Genitourinary Cancer Management*. DeKernion, J.B. & Paulson, D.F. (eds), pp 59–94. Lea & Febiger: Philadelphia.

RAGHAVAN, D. (1988). Preemptive (neo-adjuvant) intravenous chemotherapy for invasive bladder cancer. *Br. J. Urol.*, **61**, 1.

RAGHAVAN, D., PEARSON, B., DUVAL, P. & 5 others (1985). Initial intravenous cis-platinum therapy: Improved management for invasive high risk bladder cancer? *J. Urol.*, **133**, 399.

RAGHAVAN, D., GRUNDY, R., GREENAWAY, T.M. & 4 others (1988). Pre-emptive (neo-adjuvant) chemotherapy prior to radical radiotherapy for fit septuagenarians with bladder cancer: Age itself is not a contra-indication. *Br. J. Urol.*, **62**, 154.

RAGHAVAN, D., PEARSON, B., DUVAL, P. & 4 others (1989). Pre-emptive (neoadjuvant) cisplatin chemotherapy for high-risk, invasive bladder cancer. In: *Systemic Therapy for Genito-urinary Cancers*. Johnson, D.E., Logothetis, C.J. & Von Eschenbach, A.C. (eds). pp 77–84. Year Book Medical Publishers: Chicago.

RAGHAVAN, D., SHIPLEY, W.U., GARNICK, M.B., RUSSELL, P.J. & RICHIE, J.P. (1990). Biology and management of bladder cancer. *N. Engl. J. Med.* **322:** 1129.

RAGHAVAN, D., WALLACE, D.M.A., SANDEMAN, T.F. & 7 others (1989). First randomized trials of pre-emptive (neoadjuvant) intravenous cisplatin for invasive transitional cell carcinoma of bladder. *Proc. Amer. Soc. Clin. Oncol.*, **8**, 133.

ROTMAN, M., MACCHIA, R., SILVERSTEIN, M. & 4 others (1987). Treatment of advanced bladder carcinoma with irradiation and concomitant 5-fluorouracil infusion. *Cancer*, **59**, 710.

SACKS, H., BERRIER, J., REITMAN, D. & 3 others (1987). Meta-analysis of randomized controlled trials. *N. Engl. J. Med.*, **316**, 450.

SCHER, H., HERR, H., STERNBERG, C. & ? others (1989). Neo-adjuvant chemotherapy for invasive bladder cancer. Experience with the M-VAC regimen. *Br. J. Urol.*, **64**, 250.

SCHULMAN, C.C., WESPES, E., DELCOUR, C. & STRUYVEN, J. (1985). Intra-arterial chemotherapy of infiltrative bladder carcinoma. *Eur. Urol.*, **11**, 220.

SHEARER, R.J., CHILVERS, C.E.D., BLOOM, H.J.G. & 4 others (1988). Adjuvant chemotherapy in T3 carcinoma of the bladder. A prospective trial: preliminary report. *Br. J. Urol.*, **62**, 558.

SHIPLEY, W.U., COOMBS, L.J., EINSTEIN, A.B. Jr & 4 others (1984). Cisplatin and full dose irradiation for patients with invasive bladder carcinoma: A preliminary report of tolerance and local response. *J. Urol.*, **132**, 899.

SHIPLEY, W.U., KAUFMAN, S.D. & PROUT, G.R. Jr (1988). The role of radiation therapy and chemotherapy in the treatment of invasive carcinoma of the urinary bladder. *Semin. Oncol.*, **15**, 390.

SIMES, R.J. (1986). Publication bias: The case for an international registry of clinical trials. *J. Clin. Oncol.*, **4**, 1529.

SKINNER, D.G. & LEISKOVSKY, G. (1984). Contemporary cystectomy with pelvic node dissection compared to preoperative radiation therapy plus cystectomy in management of invasive bladder cancer. *J. Urol.*, **131**, 1069.

SOLOWAY, M.S., EINSTEIN, A., CORDER, M.P. & 3 others (1983). A comparison of cisplatin and the combination of cisplatin and cyclosphosphamide in advanced urothelial cancer. *Cancer*, **52**, 767.

STERNBERG, C.N., YADODA, A., SCHER, H.I. & 8 others (1985). Preliminary results of M-VAC (methotrexate, vinblastine, doxorubicin and cisplatin) for transitional cell carcinoma of the urothelium. *J. Urol.*, **133**, 403.

STERNBERG, C.N., YAGODA, A., SCHER, H.I. & 8 others (1988). M-VAC (methotrexate, vinblastine, doxorubicin and cisplatin) for advanced transitional cell carcinoma of the urothelium. *J. Urol.*, **139**, 461.

STERNBERG, C.N., YAGODA, A., SCHER, H.I. & 10 others (1989). Methotrexate, vinblastine, doxorubicin, and cisplatin for advanced transitional cell carcinoma of the urothelium: efficacy and patterns of response and relapse. *Cancer*, **64**, 2448.

TANNOCK, I.F., GOSPODAROWICZ, M., CONNOLLY, J. & 4 others (1989). M-VAC (methotrexate, vinblastine, doxorubicin, cisplatin) chemotherapy for transitional cell carcinoma: Princess Margaret Hospital experience. *J. Urol.*, **142**, 284.

TONKIN, K. & TANNOCK, I. (1988). Evaluation of response and morbidity following treatment of bladder cancer. In: *The Management of Bladder Cancer*. Raghavan, D. (ed.), pp. 228. Edward Arnold: London.

TRONER, M., BIRCH, R., OMURA, G.A. & WILLIAMS, S. (1987). Phase III comparison of cisplatin alone versus cisplatin, doxorubicin and cyclophosphamide in the treatment of bladder (urothelial) cancer: A Southeastern Cancer Study Group Trial. *J. Urol.*, **137**, 660.

VAN OOSTEROM, A.T., AKAZA, H., HALL, R. & 5 others (1986). Response criteria for phase II/III trials in invasive bladder cancer. In: *Developments in Bladder Cancer*. Denis, L, Niijima, T., Prout, G.R. Jr. & Schroeder, F.H. (eds), pp. 301. Alan R. Liss: New York.

WALLACE, D.M.A., RAGHAVAN, D., KELLY, K.A. & 6 others. Randomized trials of pre-emptive cisplatin chemotherapy for invasive bladder cancer. Submitted to *Br. J. Urology* (still pending).

WAXMAN, J., ABEL, P., JAMES, N. & ? others (1989). New combination chemotherapy programme for bladder cancer. *Br. J. Urol.*, **63**, 68.

WILSON, W.L. (1960). Chemotherapy of human solid tumours with 5-fluorouracil. *Cancer*, **13**, 1230.

YOUNG, D.C. & GARNICK, M.B. (1988). Evaluation of response and morbidity following treatment of bladder cancer. In: *The Management of Bladder Cancer*. Raghavan, D. (ed.), p. 245–263. Edward Arnold: London.

ZINCKE, H., SEN, S.E., HAHN, R.G. & KEATING, J.P. (1988). Neoadjuvant chemotherapy for locally advanced transitional cell carcinoma of the bladder: Do local findings suggest a potential for salvage of the bladder? *Mayo Clin. Proc.*, **63**, 16.